# Joint association of triglyceride glucose index (TyG) and body roundness index (BRI) with stroke incidence: a national cohort study

**DOI:** 10.1186/s12933-025-02724-6

**Published:** 2025-04-16

**Authors:** Bingxue Wang, Liying Li, Ying Tang, Xingwu Ran

**Affiliations:** 1 Department of Endocrinology and Metabolism, West China Hospital of Sichuan University, Chengdu, China; 2 Innovation Research Center for Diabetic Foot, DiabeticFootCareCenter, West China Hospital of Sichuan University, Chengdu, China; 3 Department of Cardiology, Fuwai Yunnan Cardiovascular Hospital, Kunming, China; 4 Center for High Altitude Medicine, West China Hospital, Sichuan University, Chengdu, China

**Keywords:** Triglyceride glucose index, Body roundness index, Stroke, CHARLS

## Abstract

**Background:**

Insulin resistance (IR), as quantified by the triglyceride glucose (TyG) index, and visceral obesity, as assessed by the body roundness index (BRI), have been identified as pivotal risk factors for stroke. However, the combined impact of these two indicators on stroke risk has not been thoroughly investigated. This study aims to investigate both the separate and combined associations, as well as potential interactions, between the TyG index and/or BRI with respect to stroke incidence.

**Methods:**

This cohort study encompassed 6621 respondents who were free of stroke at baseline from the China Health and Retirement Longitudinal Study (CHARLS). Participants were categorized based on the median values of the TyG index or/and BRI. Cox proportional hazards regression models were employed to examine the associations between the TyG index alone, BRI alone, and their combined effects on stroke incidence. Both additive and multiplicative interaction effects were further estimated.

**Results:**

Among 6621 participants aged 45 years or older, the mean (SD) age was 58.06 (8.57) years, with 2951 (44.6%) being male. During a follow-up period of up to 9 years, 743 individuals experienced stroke events. Compared to participants with low TyG index and low BRI, the adjusted hazard ratios (HRs) were as follows: 1.36 (95% confidence interval [CI] 1.05–1.75) for high TyG index alone, 1.61 (95% CI 1.27–2.05) for high BRI alone, and 1.78 (95% CI 1.40–2.26) for high TyG index and high BRI. Neither additive nor multiplicative interactions between BRI and TyG for incident stroke were statistically significant. The combination of TyG and BRI enhanced the predictive capability for stroke compared to either biomarker alone.

**Conclusion:**

We discovered that both the TyG index and BRI are strongly associated with stroke incidence. The joint assessment of TyG and BRI enhances the predictive capability for stroke, underscoring the critical role of IR and visceral adiposity in the identification and screening of stroke risk.

**Graphical abstract:**

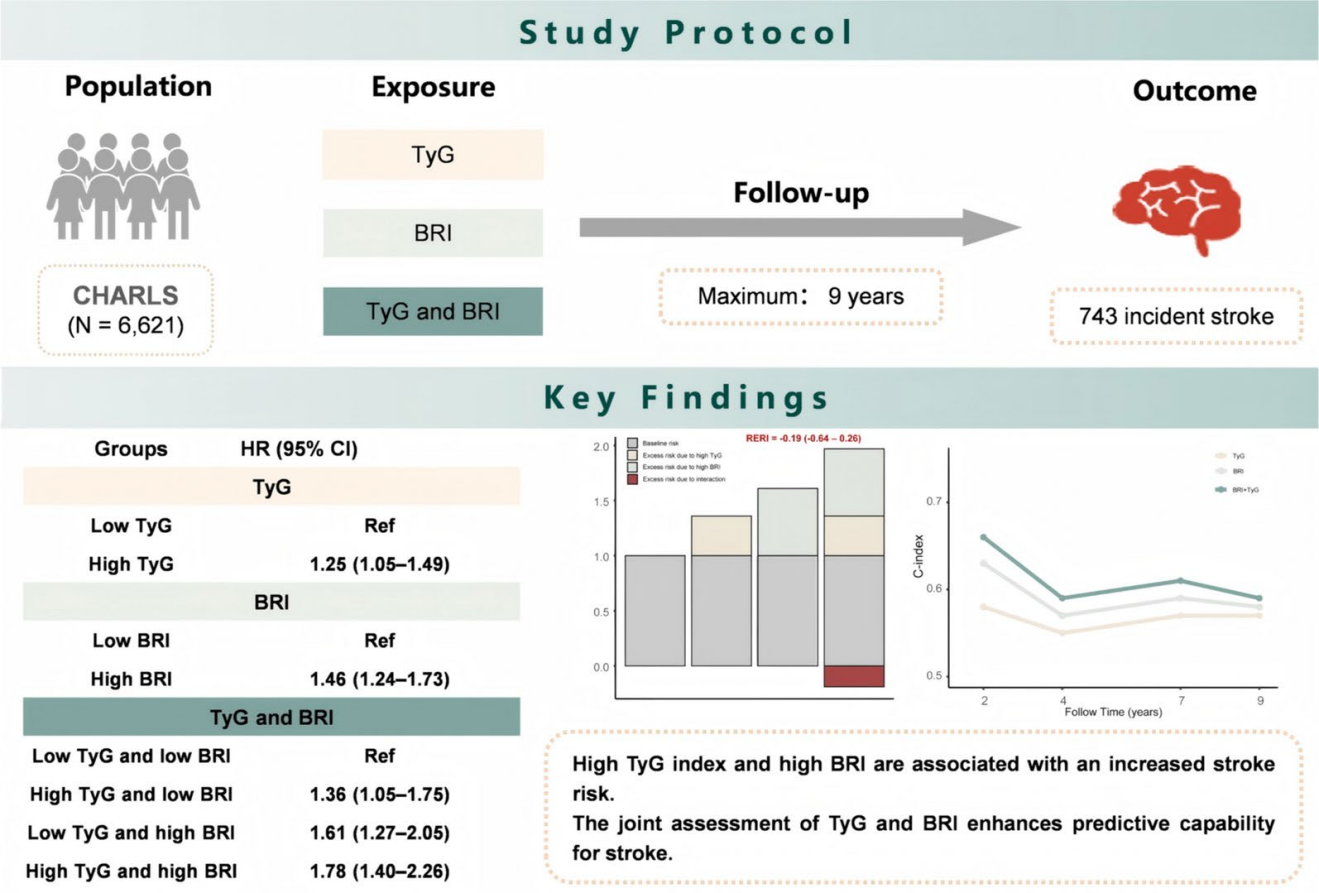

**Supplementary Information:**

The online version contains supplementary material available at 10.1186/s12933-025-02724-6.

## Introduction

Stroke remains a leading cause of mortality and disability globally, with the absolute number of stroke cases increasing substantially from 1990 to 2019 [1]. According to China Stroke Report 2021, approximately 17.8 million adults aged 40 years and older had experienced a stroke in 2020, resulting in 2.3 million fatalities [2]. Given the ongoing demographic aging and rising prevalence of metabolic diseases, the incidence of stroke is expected to continue rising [3]. Consequently, it is crucial to identify modifiable risk factors for stroke to reduce its incidence and alleviate the increasingly heavy burden.

The well-established association between insulin resistance (IR) and cardiovascular diseases (CVD) has been extensively documented. The hyperinsulinemic-euglycemic clamp test, considered the gold standard for assessing IR, is not suitable for widespread application due to its high cost and invasive nature [4]. The triglyceride-glucose (TyG) index, emerging as a reliable surrogate marker for IR [5], exhibits robust predictive power for both CVD risk factors and CVD itself [6, 7]. Obesity, particularly visceral obesity, significantly contributes to CVD risk [8, 9]. The Body Roundness Index (BRI), defined as the ratio of height to waist circumference (WC), is better to estimate the proportion of visceral fat relative to total body fat compared to traditional obesity‐related indices such as Body mass index (BMI) and WC [10]. Previous studies have demonstrated that BRI outperforms traditional anthropometric indices in predicting various health conditions, including hypertension, prediabetes, diabetes, dyslipidemia, and kidney disease [11–13]. Moreover, elevated BRI levels are strongly associated with an increased risk of CVD [14]. Notably, Obesity and IR are intricately linked, with obesity promoting IR through mechanisms such as endoplasmic reticulum stress and inflammatory responses, while IR exacerbates obesity through metabolic dysregulation [15]. Given that the TyG index provides a more comprehensive assessment of cardiovascular risk when integrated with other indicators [16–18], exploring the combined effects of the TyG index and BRI is warranted. However, evidence on the joint impact of these two indices on cardiovascular outcomes is still lacking.

To fill this knowledge gap, we utilized data from the China Health and Retirement Longitudinal Study (CHARLS), a nationally representative cohort, to investigate both the individual and joint associations of the TyG index and BRI with stroke. Additionally, we evaluate the potential interaction between these two indices. We hypothesized that the combination of the TyG index and BRI exerts a potential synergistic effect on stroke risk, demonstrating superior predictive ability compared to either index alone.

## Methods

### Study design and population

Our study utilized data from the CHARLS, an ongoing, population-based prospective cohort study designed to evaluate the health, economic, and social conditions among adults predominantly aged 45 and above [19]. Employing a multistage stratified probability sampling method, the baseline survey enrolled 17,708 participants from 150 counties or districts and 450 urban communities or villages across 28 provinces in China, ensuring a nationally representative sample. The initial survey was conducted in 2011, with follow-up investigations carried out biennially or triennially.

**Fig. 1:**
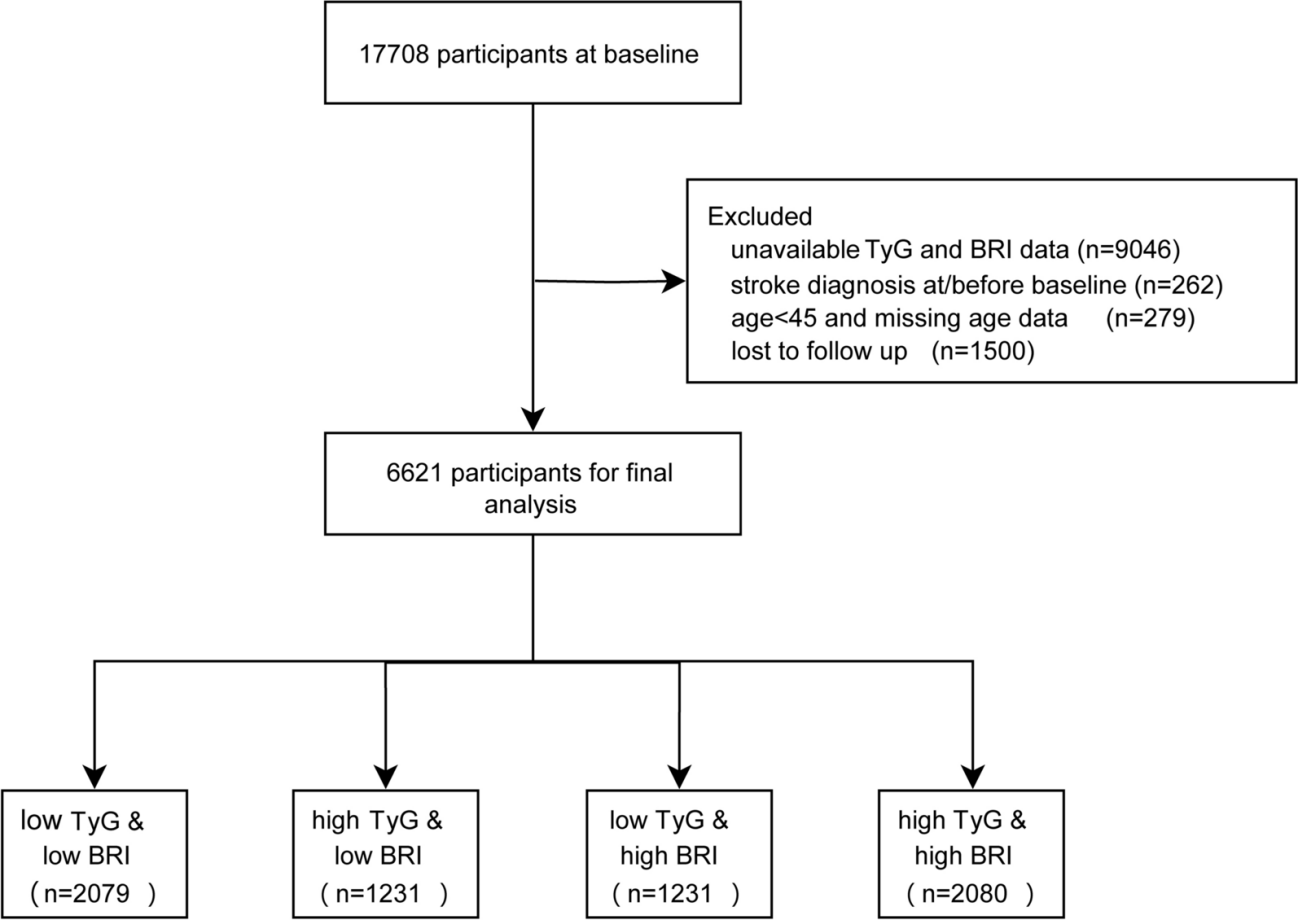
Workflow of participant recruitment and screening. Abbreviations: BRI: body roundness index; TyG: triglyceride glucose

The study encompassed a total of 17,708 participants from the baseline survey. Subsequently, 11,087 participants were excluded based on the following criteria: unavailable TyG and BRI data (n = 9046); diagnosis of stroke at or prior to the baseline survey (n = 262); age under 45 years or missing age information (n = 279); lost to follow-up (n = 1500). Finally, 6,621 participants were retained for the final analysis. Figure 1 illustrates the detailed flowchart of the inclusion and exclusion process.

## Assessment of TyG and BRI

Venous blood samples were collected from participants who had fasted for a minimum of 8 h and were subsequently transported to Beijing for measurements. Triglycerides (TG) and fasting plasma glucose (FPG) levels were measured using enzymatic colorimetric methods at the Clinical Laboratory of Capital Medical University, an institution accredited by the Beijing Health Bureau. The coefficient of variation for both TG and FPG measurements was less than 2%. The TyG index was calculated using the formula: TyG = ln[TG (mg/dL) × FPG (mg/dL)/2] [5]. Participants stood upright and barefoot on the instrument platform for height was measured using a stadiometer. WC was measured at the level of the umbilicus with participants standing. All anthropometric measurements were conducted in accordance with standardized procedures. The BRI was calculated using the formula: $${\text{BRI}} = 364.2 - 365.5 \times \sqrt {1 - \left( {\frac{{{\raise0.7ex\hbox{${{\text{WC}}}$} \!\mathord{\left/ {\vphantom {{{\text{WC}}} {2\pi }}}\right.\kern-0pt} \!\lower0.7ex\hbox{${2\pi }$}}}}{{0.5 \times {\text{height}}}}} \right)^{2} }$$ [20]

## Assessment of stroke

The primary outcome was defined as the first occurrence of a stroke event until wave 5 (2020). Stroke information was systematically collected by medically trained investigators using a standardized question: “Have you been diagnosed with a stroke by a physician?”. Subsequently, the research team conducted data verification and validation procedures to ensure accuracy.

## Covariates

Well-trained interviewers administered a structured questionnaire to systematically gather data. The collected information was categorized into several domains: sociodemographic characteristics (age, sex, place of residence, marital status, educational background); lifestyle factors (smoking and drinking); anthropometric measurements (BMI, systolic blood pressure [SBP], and diastolic blood pressure [DBP]); medical history (diabetes, hypertension, hyperlipidemia, kidney disease, heart disease); treatments (antihyperglycemic agents, antihypertensive medications, and lipid-lowering therapies); and laboratory tests results (glycosylated hemoglobin A1c [HbA1c], total cholesterol [TC], TG, high-density lipoprotein cholesterol [HDL-C], low-density lipoprotein cholesterol [LDL-C], uric acid [UA], and C-reactive protein [CRP]).

The BMI was calculated using the formula: BMI (kg/m^2^) = body mass(kg)/height(m)^2. Diabetes mellitus was diagnosed based on self-reported diabetes history, use of antidiabetic medications, FPG ≥ 7.0 mmol/L, or HbA1c levels ≥ 6.5% [21]. Hypertension was defined as a self-reported hypertension history, use of antihypertensive drugs, SBP ≥ 140 mmHg, or DBP ≥ 90 mmHg [22]. Dyslipidemia was identified via self-reported dyslipidemia history, use of lipid-lowering medications, or laboratory tests indicating TG ≥ 150 mg/dL, TC ≥ 240 mg/dL, HDL-C < 40 mg/dL, or LDL-C ≥ 160 mg/dL [23].

## Statistical analysis

The Kolmogorov–Smirnov test and Levene's test were employed to evaluate the normality of distributions and homogeneity of variance for continuous variables, respectively. Continuous variables adhering to normal distributions were expressed as means with standard deviations (SD), whereas those exhibiting skewed distributions were presented as medians with interquartile ranges (IQR). Categorical data were summarized using frequencies and percentages. In accordance with previous literature [16–18], the median values of TyG (8.58) and BRI (4.05) were employed as cutoff points to classify respondents into four categories: low TyG and low BRI, high TyG and low BRI, low TyG and high BRI, high TyG and high BRI. The differences among these groups were examined using the Kruskal–Wallis test for continuous variables and the chi-square test for categorical variables.

Multiple imputation by chained equations (MICE) was performed to address the 5% missing data (341 out of 6,621 subjects), assuming that the data were missing at random. Detailed summaries of the missing data counts and the specific imputation methods employed are provided in Supplementary Table S1.

The stroke incidence rate per 1000 person-years was estimated. Kaplan–Meier curves were illustrated to estimate the cumulative incidence of stroke, and differences between groups were assessed using the log-rank test. As no evidence of proportional hazards assumption violation was observed, crude, partially adjusted, and fully adjusted Cox proportional hazards models were fitted to investigate the associations between the TyG index and BRI with new-onset stroke. Subsequently, multiplicative interaction was evaluated by incorporating a cross-product term between TyG and BRI into the Cox model. Interaction on the additive scale was assessed using three indices [24]: (1) The relative excess risk due to interaction (RERI): RERI quantifies the additional risk attributable to interaction by calculating the difference between the joint effect and the sum of the individual effects. (2) The attributable proportion due to interaction (AP): AP implies the proportion of the outcome that can be attributed to the interaction between the both exposures. (3) The synergy index (SI): SI reflects the ratio of the excess risk associated with both exposures to the sum of the increased risks due to separate exposure. Additionally, the time-dependent Harrell’s C-index was computed to assess the predictive performance of TyG, BRI, and their combination for stroke incidence.

Subgroup analyses were conducted to investigate potential heterogeneity in the association between the TyG index, BRI, and their joint effect on stroke incidence. Sensitivity analyses were performed by excluding those with incomplete covariate data, those receiving antihyperglycemic, antihypertensive, or lipid-lowering treatments at baseline, those with a follow-up duration of less than two years, and by including those with non-fasting blood samples.

All statistical analyses were conducted using R software, version 4.3.0. Two-sided *P*-values less than 0.05 indicate statistical significance.

## Results

### Baseline characteristics of participants

A total of 6,621 participants (44.6% male) were included in this study, with an average age of 58.06 ± 8.57 years. Participants were categorized into four subgroups based on TyG and BRI levels: low TyG and low BRI (n = 2079), high TyG and low BRI (n = 1231), low TyG and high BRI (n = 1231), and high TyG and high BRI (n = 2080). The baseline characteristics of the participants were summarized in Table 1. Statistically significant differences were observed in most baseline characteristics among the four subgroups, including age, sex, place of residence, smoking status, drinking, BMI, SBP, DBP, prevalence of hypertension, diabetes, hyperlipidemia, heart diseases, and treatments for hyperglycemia, hypertension, and dyslipidemia. Additionally, statistically significant differences were noted in TC, TG, LDL-C, HDL-C, UA, and CRP. The baseline characteristics of participants according to the median values of TyG and BRI are presented in Supplementary Tables S2 and S3.

**Table 1:** Baseline characteristics of participants

Variables	All	Low TyG and low BRI	High TyG and low BRI	Low TyG and high BRI	High TyG and high BRI	P value
Number of participants	6621	2079	1231	1231	2080	
Age, years, mean (SD)	58.06 (8.57)	57.74 (8.59)	57.45 (8.22)	58.39 (8.94)	58.56 (8.49)	< 0.001
Male, n (%)	2951 (44.6)	1238 (59.5)	664 (53.9)	383 (31.1)	666 (32.0)	< 0.001
Residence, n (%)						< 0.001
Rural	2228 (33.7)	576 (27.7)	381 (31.0)	406 (33.0)	865 (41.6)	
City	4393 (66.3)	1503 (72.3)	850 (69.0)	825 (67.0)	1215 (58.4)	
Marital status, n (%)						0.688
Married and living with spouse	966 (14.6)	299 (14.4)	178 (14.5)	193 (15.7)	296 (14.2)	
Others	5655 (85.4)	1780 (85.6)	1053 (85.5)	1038 (84.3)	1784 (85.8)	
Education level, n (%)						0.146
Junior high school and below	5995 (90.5)	1874 (90.1)	1098 (89.2)	1127 (91.6)	1896 (91.2)	
Senior high school and above	626 (9.5)	205 (9.9)	133 (10.8)	104 (8.4)	184 (8.8)	
Smoking, n (%)	2450 (37.1)	1013 (48.8)	554 (45.0)	317 (25.8)	566 (27.3)	< 0.001
Drinking, n (%)	2539 (38.4)	963 (46.3)	534 (43.4)	394 (32.0)	648 (31.2)	< 0.001
BMI, kg/m^2^	23.2 (21.0, 25.8)	21.0 (19.5, 22.5)	21.6 (20.0, 23.1)	25.0 (23.2, 26.9)	26.1 (24.2, 28.2)	< 0.001
SBP, mmHg	126.3 (114.3, 140.7)	120.7 (110.0, 133.3)	125.0 (113.0, 137.3)	128.3 (116.0, 143.7)	132.3 (119.5, 147.3)	< 0.001
DBP, mmHg	74.7 (67.3, 83.0)	71.3 (64.3, 79.3)	74.0 (66.7, 81.7)	75.7 (68.7, 84.0)	78.0 (70.3, 86.3)	< 0.001
Antihyperglycemic treatment, n (%)	225 (3.4)	22 (1.1)	39 (3.2)	27 (2.2)	137 (6.7)	< 0.001
Antihypertensive treatment, n (%)	1212 (18.4)	176 (8.5)	168 (13.8)	233 (19.0)	635 (30.6)	< 0.001
Lipid-lowering treatment, n (%)	323 (5.0)	33 (1.6)	31 (2.6)	54 (4.5)	205 (10.1)	< 0.001
Diabetes, n (%)	992 (15.2)	102 (5.0)	240 (19.8)	89 (7.4)	561 (27.3)	< 0.001
Hypertension, n (%)	2620 (40.0)	503 (24.5)	421 (34.6)	547 (44.8)	1149 (55.6)	< 0.001
Hyperlipidemia, n (%)	3142 (48.0)	383 (18.7)	804 (66.2)	367 (30.2)	1588 (76.9)	< 0.001
Kidney disease, n (%)	354 (5.4)	118 (5.7)	61 (5.0)	70 (5.7)	105 (5.1)	0.700
Heart disease, n (%)	701 (10.6)	152 (7.3)	112 (9.2)	120 (9.8)	317 (15.3)	< 0.001
TC, mg/dL	191.8 (168.9, 216.1)	180.9 (161.2, 204.1)	197.6 (174.0, 222.9)	187.5 (166.6, 209.2)	202.0 (178.5, 228.1)	< 0.001
TG, mg/dL	103.5 (74.3, 150.5)	71.7 (57.5, 86.7)	143.4 (121.3, 188.5)	79.7 (64.6, 92.9)	156.7 (125.7, 215.3)	< 0.001
HDL-C, mg/dL	49.5 (40.6, 59.9)	57.6 (49.1, 68.0)	46.0 (38.3, 55.7)	53.4 (45.2, 63.0)	42.5 (35.2, 49.9)	< 0.001
LDL-C, mg/dL	116.0 (95.1, 138.4)	109.8 (91.2, 129.5)	116.8 (95.5, 140.3)	117.5 (100.1, 138.4)	122.2 (97.4, 145.8)	< 0.001
UA, mg/dL	4.3 (3.5, 5.1)	4.2 (3.5, 4.9)	4.3 (3.6, 5.2)	4.0 (3.4, 4.8)	4.4 (3.7, 5.3)	< 0.001
CRP, mg/L	1.0 (0.5, 2.0)	0.7 (0.4, 1.5)	0.9 (0.5, 1.9)	1.0 (0.5, 2.1)	1.34 (0.8, 2.6)	< 0.001
BRI	4.05 (3.24, 5.08)	3.16 (2.69, 3.58)	3.37 (2.89, 3.71)	4.92 (4.46, 5.60)	5.19 (4.64, 6.00)	< 0.001
TyG	8.58 (8.22, 9.01)	8.17 (7.95, 8.38)	8.93 (8.74, 9.24)	8.28 (8.08, 8.44)	9.06 (8.80, 9.48)	< 0.001
Stroke, n (%)	743 (11.2)	141 (6.8)	129 (10.5)	148 (12.0)	325 (15.6)	< 0.001

Values are mean (SD), median (IQR), or n (%)

Abbreviations: BRI: body roundness index; BMI: body mass index; CRP: C-reactive protein; DBP: diastolic blood pressure; HDL-C: high-density lipoprotein cholesterol; IQR: Interquartile range; LDL-C: low-density lipoprotein cholesterol; SBP: systolic blood pressure; SD: standard deviation; TC: total cholesterol; TG: triglycerides; TyG: triglyceride glucose; UA: uric acid

## Association of TyG and BRI with the risk of stroke

**Fig. 2:**
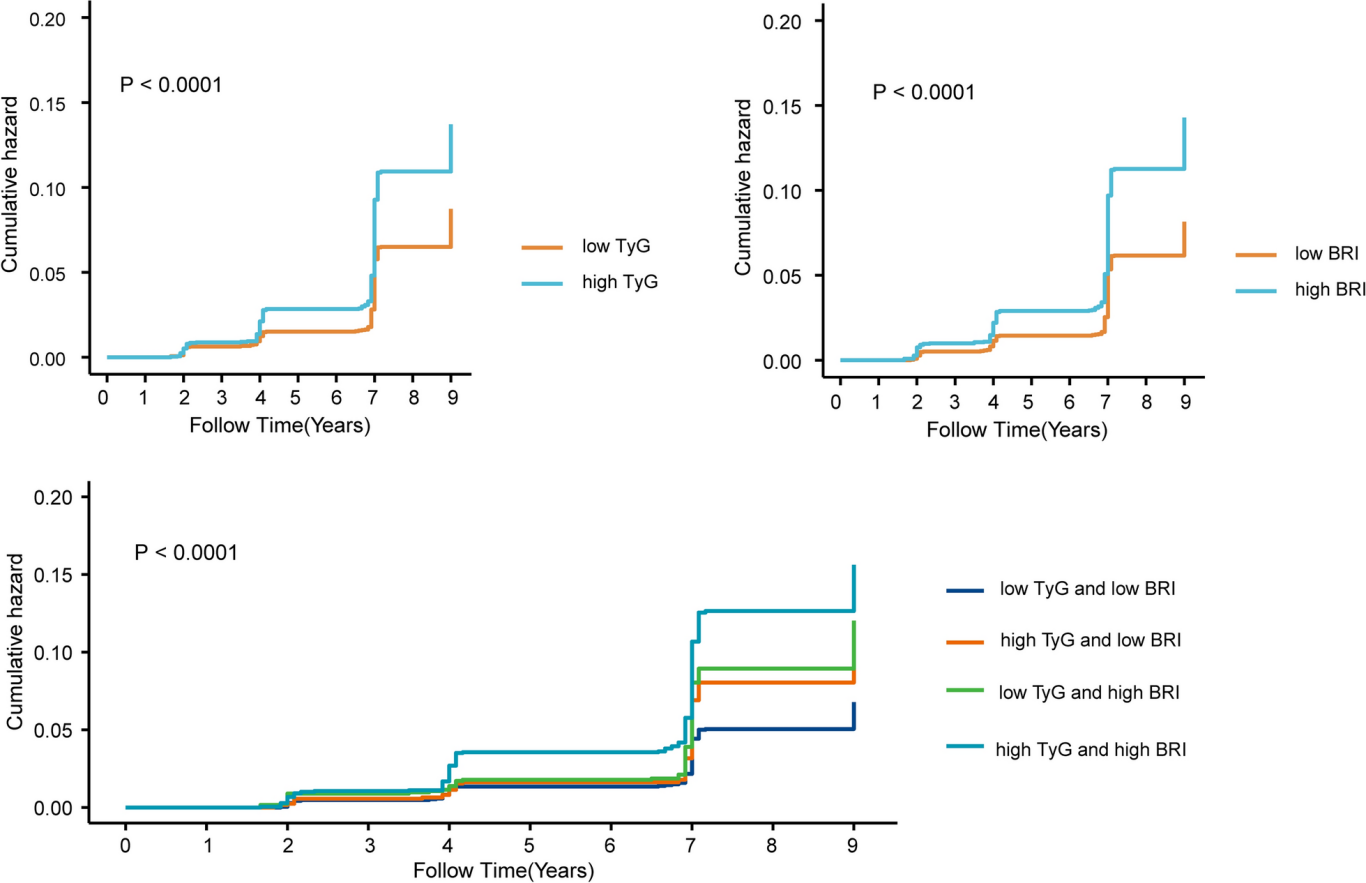
Kaplan–Meier plot of stroke by TyG index and BRI level. Abbreviations: BRI: body roundness index; TyG: triglyceride glucose

During a maximum follow-up period of 9 years, 743 participants experienced incident stroke. Kaplan–Meier survival curves demonstrated that the cumulative incidence of stroke increased with high BRI, high TyG, and both high BRI and high TyG (Fig. 2) (all log-rank *P* < 0.05). Cox proportional hazards regression analyses with different adjustments, summarized in Table 2, were conducted to evaluate the associations between TyG, BRI, and the risk of stroke. After adjusting for potential confounding variables (Model 3), participants with high TyG exhibited a 1.25-fold increased risk of stroke (95% confidence interval [CI]: 1.05–1.49) in comparison to those with low TyG. Similarly, respondents with high BRI exhibited a 1.46-fold increased risk of stroke (95% CI: 1.24–1.73) relative to those with low BRI. Taking participants with low TyG and low BRI as the reference group, individuals with solely high TyG, solely high BRI, and both high TyG and high BRI were significantly associated with elevated risks of stroke by 36% (95% CI 1.05–1.75), 61% (95% CI 1.27–2.05), and 78% (95% CI: 1.40–2.26), respectively. Additionally, we assessed the predictive performance of TyG and BRI for stroke at each wave, revealing that the combination of TyG and BRI provided the highest predictive capability for stroke (Supplementary Fig. S1).

**Table 2:** Association of TyG and BRI and the risk of stroke incidence

	Case	Incidence rate^a^ (95% CI)	Crude model		Model 1		Model 2		Model 3	
			HR (95% CI)	P value	HR (95% CI)	P value	HR (95% CI)	P value	HR (95% CI)	P value
TyG										
Low TyG	289	9.9 (8.8–11.1)	Ref		Ref		Ref		Ref	
High TyG	454	15.8 (14.4–17.3)	1.62 (1.40–1.87)	< 0.001	1.66 (1.43–1.92)	< 0.001	1.52 (1.30–1.76)	< 0.001	1.25 (1.05–1.49)	0.015
BRI										
Low BRI	270	9.2 (8.2–10.4)	Ref		Ref		Ref		Ref	
High BRI	473	16.5 (15.1–18.0)	1.82 (1.56–2.11)	< 0.001	1.92 (1.64–2.24)	< 0.001	1.71 (1.46–2.00)	< 0.001	1.46 (1.24–1.73)	< 0.001
TyG and BRI										
Low TyG and low BRI	141	7.7 (6.5–9.1)	Ref		Ref		Ref		Ref	
High TyG and low BRI	129	11.9 (10.0–14.2)	1.57 (1.24–2.00)	< 0.001	1.64 (1.29–2.09)	< 0.001	1.56 (1.23–1.98)	< 0.001	1.36 (1.05–1.75)	0.019
Low TyG and high BRI	148	13.7 (11.7–16.2)	1.82 (1.45–2.29)	< 0.001	1.95 (1.54–2.47)	< 0.001	1.78 (1.40–2.25)	< 0.001	1.61 (1.27–2.05)	< 0.001
High TyG and high BRI	325	18.1 (16.3–20.2)	2.43 (1.99–2.96)	< 0.001	2.62 (2.14–3.21)	< 0.001	2.26 (1.84–2.79)	< 0.001	1.78 (1.40–2.26)	< 0.001

^a^ Per 1000 person-years

Model 1: adjusted for age and sex

Model 2: adjusted for variables in Model 1 plus smoking, drinking, marital status, education level, residence, and SBP

Model 3: adjusted for variables in Model 2 plus heart disease, diabetes, hypertension, hyperlipidemia, kidney disease, C-reactive protein, and uric acid

Abbreviations: BRI: body roundness index; TyG: triglyceride glucose

## Interaction between TyG and BRI

Following a comprehensive adjustment for potential confounders, we observed that the 95% confidence intervals for RERI and AP included 0, whereas those for SI and the multiplicative effect included 1, indicating an absence of statistically significant additive or multiplicative interactions between TyG and BRI regarding stroke (Table 3).

**Table 3:** Interaction between the TyG index and BRI on stroke risk

Interactive indices	Interactive effects (95% CI)		
	Model 1	Model 2	Model 3
Additive effect			
RERI	0.03 (− 0.50–0.56)	− 0.07 (− 0.57–0.43)	− 0.19 (− 0.64–0.26)
AP	0.01 (− 0.19–0.21)	− 0.03 (− 0.25–0.19)	− 0.11 (− 0.36–0.14)
SI	1.02 (0.73–1.42)	0.95 (0.65–1.38)	0.80 (0.50–1.28)
Multiplicative effect	0.82 (0.60–1.11)	0.82 (0.60–1.11)	0.81 (0.60–1.11)

Model 1: adjusted for age and sex

Model 2: adjusted for variables in Model 1 plus smoking, drinking, marital status, education level, residence, and SBP

Model 3: adjusted for variables in Model 2 plus heart disease, diabetes, hypertension, hyperlipemia, kidney disease, C-reactive protein, and uric acid

Abbreviations: AP: proportion attributable to interaction; BRI: body roundness index; CI: confidence interval; TyG: triglyceride glucose; RERI: relative excess risk due to interaction; SI: synergy index

## Subgroup analyses

We performed stratified analyses to assess the associations of BRI and TyG with stroke events across various subgroups. The associations between BRI and TyG and the risk of stroke in most subgroups were consistent with the main results. No significant interaction was observed (Table 4). Similar results were observed when stratified by BRI (Supplementary Fig. S3). Notably, a significant interaction was observed between sex and TyG regarding stroke risk (P for interaction = 0.049). Specifically, a high TyG index was more strongly associated with an elevated risk of stroke in males than in females (Supplementary Fig. S2).

**Table 4:** Subgroup analysis for the association of the TyG index and BRI on stroke risk

	Low TyG and low BRI	High TyG and low BRI	Low TyG and High BRI	High TyG and high BRI	*P* for interaction
Age (years)					0.808
< 60	Ref	1.41 (1.00–2.03)	1.56 (1.08–2.24)	1.82 (1.28–2.58)	
≥ 60	Ref	1.35 (0.95–1.94)	1.67 (1.21–2.30)	1.71 (1.24–2.38)	
Sex					0.505
Female	Ref	1.26 (0.84–1.91)	1.53 (1.08–2.17)	1.73 (1.21–2.46)	
Male	Ref	1.35 (0.97–1.87)	1.54 (1.10–2.16)	1.80 (1.30–2.48)	
Smoking					0.787
No	Ref	1.25 (0.86–1.83)	1.54 (1.11–2.14)	1.84 (1.33–2.56)	
Yes	Ref	1.38 (0.97–1.95)	1.63 (1.14–2.33)	1.64 (1.16–2.33)	
Drinking					0.853
No	Ref	1.41 (0.97–2.03)	1.79 (1.29–2.49)	1.98 (1.42–2.76)	
Yes	Ref	1.32 (0.92–1.89)	1.42 (0.99–2.03)	1.59 (1.13–2.25)	
Residence					0.938
City	Ref	1.60 (0.99–2.59)	1.73 (1.11–2.70)	1.97 (1.28–3.03)	
Rural	Ref	1.30 (0.96–1.75)	1.59 (1.20–2.12)	1.72 (1.29–2.29)	
Education					0.273
Junior high school and below	Ref	1.29 (0.99–1.68)	1.49 (1.16–1.91)	1.65 (1.29–2.12)	
Senior high school and above	Ref	2.84 (1.07–7.56)	4.02 (1.61–10.01)	4.16 (1.61–10.71)	
Diabetes					0.172
No	Ref	1.44 (1.09–1.89)	1.66 (1.30–2.13)	1.68 (1.30–2.18)	
Yes	Ref	1.20 (0.53–2.71)	1.22 (0.47–3.12)	2.10 (0.98–4.52)	
Hypertension					0.639
No	Ref	1.38 (0.96–1.99)	1.76 (1.24–2.50)	1.69 (1.18–2.43)	
Yes	Ref	1.39 (0.96–2.00)	1.52 (1.09–2.13)	1.80 (1.29–2.49)	
Dyslipidemia					0.246
No	Ref	1.58 (1.09–2.31)	1.76 (1.31–2.36)	2.09 (1.49–2.93)	
Yes	Ref	1.03 (0.71–1.50)	1.20 (0.79–1.83)	1.39 (0.98–1.97)	

Abbreviations: BRI: body roundness index; TyG: triglyceride glucose

## Sensitivity analyses

**Fig. 3:**
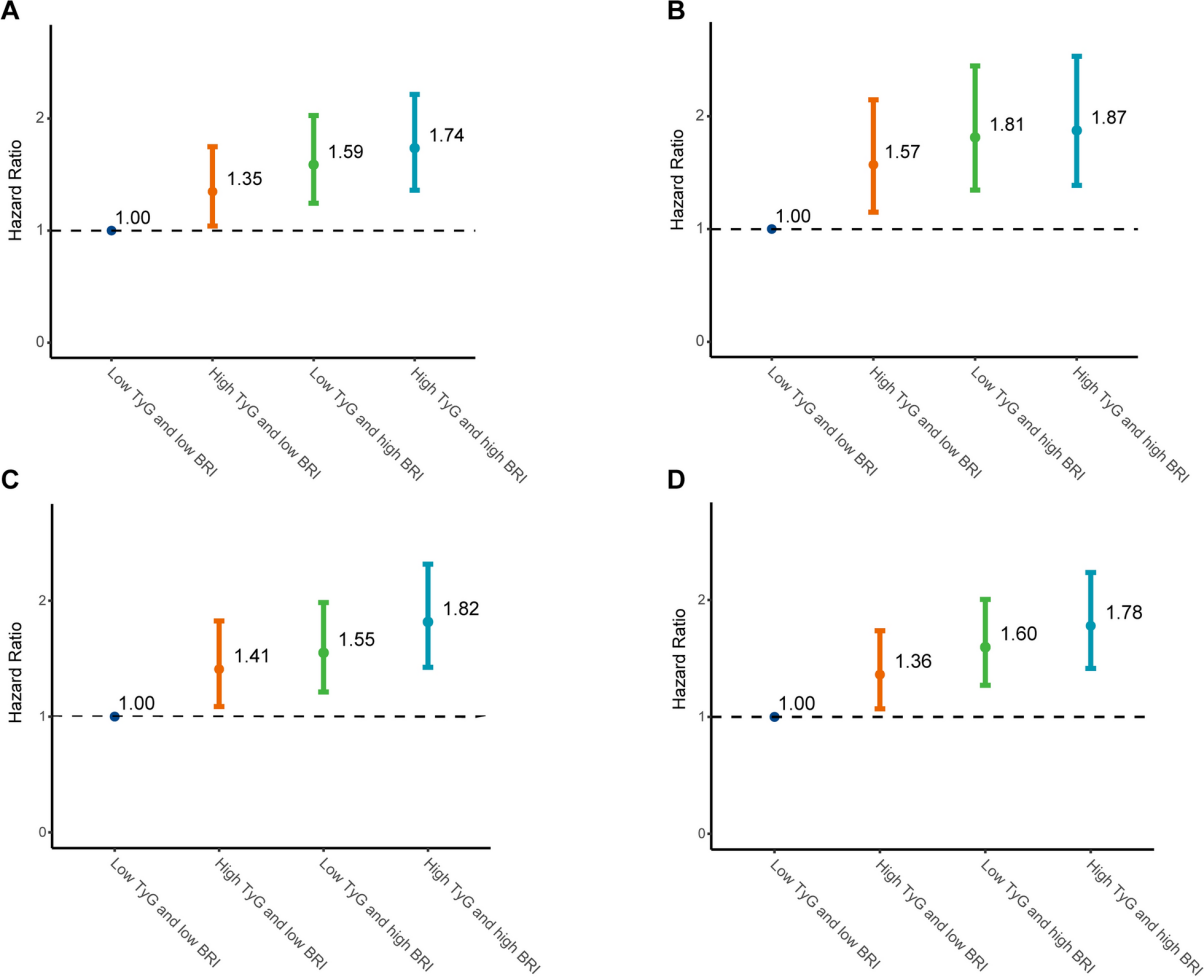
Sensitivity analysis of the combined effect of the TyG index and BRI level on stroke risk. (**A**) excluding participants with missing covariate data; (**B**) excluding participants receiving antihyperglycemic treatment or antihypertensive treatment or lipid-lowering treatment; (**C**) excluding participants with follow duration less than two years; (**D**) including participants with non-fasting blood samples Adjusted for age, sex, smoking, drinking, marital status, education level, residence, SBP, heart disease, diabetes, hypertension, hyperlipemia, kidney disease, C-reactive protein, and uric acid. Abbreviations: BRI: body roundness index; TyG: triglyceride glucose

Several sets of sensitivity analyses were conducted to assess the robustness of the main findings (Supplementary Table S5 and Fig. 3). The associations of TyG and BRI with the risk of stroke remained consistent with the main findings after excluding participants with missing data. This association also remained unchanged after removing participants receiving antihyperglycemic, antihypertensive, and lipid-lowering treatments at baseline. The results persisted even after excluding participants with a follow-up duration of less than 2 years. Additionally, including participants with non-fasting blood samples at baseline did not compromise the stability of the results.

## Discussion

We observed that the high TyG index and high BRI were significantly associated with an elevated risk of stroke in a representative sample of 6,621 individuals. When participants were stratified by the median values of the TyG index and BRI, those with concurrently high TyG index and high BRI exhibited the highest risk of stroke, with a 78% increased risk compared to those with low levels of both indices. No significant interaction was identified between TyG and BRI on stroke risk. Additionally, subgroup analyses and sensitivity analyses consistently supported the primary findings. 

The TyG index is widely acknowledged as a reliable and cost-effective surrogate marker for IR [5, 25]. Our study confirms the positive association between the TyG index and stroke events, aligning with prior meta-analyses [26, 27]. The TyG index has also been demonstrated to be associated with adverse stroke prognosis, including increased in-hospital mortality, diminished long-term survival rates, and a heightened risk of stroke recurrence [28–31]. Mechanistically, IR induces endothelial dysfunction, smooth muscle cell proliferation, inflammation and oxidative stress, all of which can accelerate the formation and progression of atherosclerosis [32]. Additionally, IR contributes to decreased the secretion of nitric oxide by inhibiting the PI3-K pathway, thereby promoting vasoconstriction [33]. Accompanied by elevated aldosterone levels, IR leads to arterial stiffness via activation of the endothelial Na^+^ channel [34]. Individuals with IR are more prone to thrombus formation due to platelet over-activation, upregulation of coagulation system, and downregulation of fibrinolytic activity [35]. More importantly, high TyG levels exhibit a strong correlation with well-established risk factors for stroke, such as metabolic disorders, kidney disease and atrial fibrillation [7, 36, 37]. Subgroup analyses revealed a significant interaction between sex and the TyG index. Specifically, in the male subgroup, a high TyG index was associated with a 1.32-fold increased risk of new-onset stroke compared to a low TyG index. In contrast, this association was relatively attenuated in the female subgroup. Dang et al. and Guo et al. reported that the association between the TyG index and CVD, as well as impaired cardiovascular fitness, was more pronounced in men [38, 39]. Conversely, Yan et al. found no significant interaction between the TyG index and sex regarding stroke events [27]. The discrepancies in these findings may partly stem from variations in study populations. In addition to this, the protective effects of estrogen in female may play a role [40]. Another plausible explanation is that unadjusted high-risk lifestyle factors, including excessive salt intake and sedentary behavior, could amplify the impact of the TyG index in men [41]. Considering the protective effects of antihypertensive, lipid-lowering, and antidiabetic medications [42–44], we performed additional sensitivity analyses by excluding individuals receiving these treatments. The results remained consistent, thereby affirming the prognostic value of the TyG index for predicting stroke risk.

BRI, developed by Thomas et al. in 2013, has been recognized as a superior indicator for evaluating abdominal obesity [10]. A cohort study involving 15,848 patients with diabetes or prediabetes revealed a non-linear relationship between BRI and cardiovascular mortality after nearly 8 years of follow-up. Specifically, when BRI exceeds 5.21, the risk of CVD increases significantly by 13% [45]. The Tehran Lipid and Glucose Study, which followed 6,840 females over a period of up to 16 years, found that BRI was associated with a 60% increased risk of CVD among postmenopausal women at baseline or those who transitioned to postmenopausal status during the study [46]. Additionally, Yang et al. utilized latent class mixed models to simulate BRI trajectories. Compared to participants with low-stable BRI trajectories, those with moderate-stable and high-stable BRI trajectories exhibited a 29% and 46% higher risk of stroke, respectively [20]. Our findings indicate that individuals with a BRI exceeding 4.3 are significantly associated with a 46% increased risk of stroke. Consequently, incorporating BRI assessment into clinical practice can enhance stroke risk stratification and facilitate more targeted preventive strategies. The mechanisms linking the BRI to stroke can be elucidated by visceral fat accumulation, which triggers a cascade of responses, including increased production of reactive oxygen species, elevated levels of pro-inflammatory cytokines, dysregulation of adipokine secretion, hypoxia, and IR. These processes collectively impair the structure and function of the cardiovascular system, ultimately contributing to the development of stroke [47–49].

Evidence demonstrated that TyG or BRI, when combined with other biomarkers, captures the risk of CVD more effectively [14, 16–18]. However, studies integrating TyG with BRI remain limited. A study by Yao et al. indicated that elevated TyG-BRI correlates with a 15% increased risk of ischemic stroke [50]. In our study, using low TyG and low BRI as the reference group, we observed that elevated BRI alone was associated with a 36% increased risk of stroke. Similarly, elevated TyG alone corresponded to a 61% higher risk. Notably, the coexistence of high BRI and high TyG was linked to a 78% higher risk of stroke. In contrast to Yao et al.'s study, which was confined to rural residents in northeastern China, our research recruited participants from diverse urban and rural areas across the country, yielding a more representative sample and significantly enhancing the generalizability of our findings. Additionally, we compared the predictive power of the combined TyG and BRI index relative to that of TyG and BRI individually across different survey periods. The combined TyG and BRI index significantly enhanced stroke prediction when analyzed as categorical variables, whereas the improvement was relatively modest when analyzed as continuous variables. Both TyG and BRI are non-invasive markers that can be easily obtained in clinical practice. Incorporating these indices into clinical settings could offer a novel and practical approach for screening and intervening with individuals at high risk of stroke.

IR is closely associated with abnormal lipid metabolism, resulting in elevated levels of free fatty acids. Once the storage capacity of subcutaneous adipose tissue is exceeded, lipids accumulate within the abdominal cavity [51]. Hepatic lipid accumulation damages glucose metabolism by stimulating glucose production and reducing insulin degradation, thereby elevating insulin levels [52]. Intrapancreatic fat deposition further impairs glucose metabolism and leads to dysfunction in insulin secretion [53]. Adipose tissue dysregulates adipokine secretion and promotes overproduction of pro-inflammatory factors, characterized by increased levels of tumor necrosis factor-α and interleukin-6, as well as decreased levels of adiponectin, all of which exacerbate IR [54, 55]. Theoretically, IR and visceral fat mutually reinforce each other, forming a vicious cycle [56]. In our study, the evidence is insufficient to demonstrate either additive or multiplicative interaction between TyG and BRI concerning stroke events. Previous studies have indicated that neither BMI nor Chinese visceral adiposity index (CVAI) interacts significantly with TyG in relation to stroke risk [57, 58]. Visceral fat increases progressively with age, thereby exacerbating IR and increasing the burden on pancreatic islet cells [59, 60]. This condition worsens with the islet β-cell aging [61, 62], ultimately leading to decreased insulin secretion, especially in individuals with baseline deficiencies in pancreatic islet function. Metabolic disorders associated with elevated insulin levels may be alleviated. Yang et al. reported that a high TyG index plays a protective role in groups with high CVAI [58]. The dynamic changes in visceral fat and IR add complexity to the exploration of interactions. Moreover, the TyG-BRI index exhibits a significant protective effect on hemorrhagic stroke within a certain range, which is not observed in ischemic stroke [50]. Given the distinct pathogenesis underlying different stroke subtypes, further research is essential to unravel the potential interaction between TyG and BRI across stroke subtypes.

Several limitations of this study warrant consideration. Firstly, due to the observational nature of the study design, residual confounding factors such as psychological traits, household income, and physical activity may still influence the results, and the causal relationships between TyG, BRI, and stroke cannot be ascertained. Secondly, in the absence of standardized clinical cut-off values for BRI and TyG, participants were categorized based on median values, which may not adequately reflect clinically relevant thresholds. Future research is needed to establish more precise grouping criteria. Thirdly, the stroke outcome was determined based on self-reported physician diagnosis, introducing potential recall bias and misclassification bias. In addition, the stroke information was relatively preliminary. This limitation impedes an in-depth analysis of the relationships between TyG, BRI, and specific stroke subtypes (e.g., ischemic stroke, hemorrhagic stroke). Changes in medication use during the follow-up period were not considered, despite reanalyzing the dataset after excluding participants who received glucose-lowering, blood pressure-lowering, and lipid-lowering treatments at baseline. It is noteworthy that the CHARLS survey specifically targeted middle-aged and older individuals in China [19], thus highlighting the necessity for future research to validate the generalizability of these findings across diverse populations and cultural contexts.

## Conclusion

Our findings demonstrate that elevated TyG and BRI are significantly correlated with an increased risk of stroke. Although no synergistic effect between TyG and BRI was observed, the concurrent evaluation of TyG and BRI indices enhances the predictive capacity for stroke events. supporting the role of IR and visceral adiposity in identifying and screening individuals at risk of stroke.

## Supplementary Information

Supplementary material 1

## Data Availability

No datasets were generated or analysed during the current study.

## Abbreviations

AP: Attributable proportion due to interaction
BMI: Body mass index
BRI: Body roundness index
CHARLS: China Health and Retirement Longitudinal Study
CI: Confidence interval
CRP: C-reactive protein
CVD: Cardiovascular diseases
CVAI: Chinese visceral adiposity index
DBP: Diastolic blood pressure
FPG: Fasting plasma glucose
HbA1c: Glycosylated hemoglobin A1c
HDL-C: High density lipoprotein cholesterol
HR: Hazard ratio
IQR: Interquartile range
IR: Insulin resistance
LDL-C: Low density lipoprotein cholesterol
MICE: Multiple Imputation by Chained Equations
RERI: Relative excess risk due to interaction
SBP: Systolic blood pressure
SD: Standard deviation
SI: Synergy index
TC: Total cholesterol
TG: Triglyceride
TyG: Triglyceride glucose
UA: Uric acid
WC: Waist circumference

## References

[1] GBD 2021 Stroke Risk Factor Collaborators. Global, regional, and national burden of stroke and its risk factors, 1990–2021: a systematic analysis for the Global Burden of Disease Study 2021. Lancet Neurol 2024; 23(10):973–1003.10.1016/S1474-4422(24)00369-739304265

[2] Tu WJ, Wang LD. China stroke surveillance report 2021. Mil Med Res. 2023;10(1):33. 37468952 10.1186/s40779-023-00463-x PMC10355019

[3] Cheng Y, Lin Y, Shi H, Cheng M, Zhang B, Liu X, et al. Projections of the Stroke Burden at the Global, Regional, and National Levels up to 2050 Based on the Global Burden of Disease Study 2021. J Am Heart Assoc. 2024;13(23): e036142. 39575720 10.1161/JAHA.124.036142 PMC11681572

[4] DeFronzo RA, Tobin JD, Andres R. Glucose clamp technique: a method for quantifying insulin secretion and resistance. Am J Physiol. 1979;237(3):E214-223. 382871 10.1152/ajpendo.1979.237.3.E214

[5] Tahapary DL, Pratisthita LB, Fitri NA, Marcella C, Wafa S, Kurniawan F, et al. Challenges in the diagnosis of insulin resistance: Focusing on the role of HOMA-IR and Tryglyceride/glucose index. Diabetes Metab Syndr. 2022;16(8): 102581. 35939943 10.1016/j.dsx.2022.102581

[6] Avagimyan A, Pogosova N, Fogacci F, Aghajanova E, Djndoyan Z, Patoulias D, et al. Triglyceride-glucose index (TyG) as a novel biomarker in the era of cardiometabolic medicine. Int J Cardiol. 2025;418: 132663. 39426418 10.1016/j.ijcard.2024.132663

[7] Gounden V, Devaraj S, Jialal I. The role of the triglyceride-glucose index as a biomarker of cardio-metabolic syndromes. Lipids Health Dis. 2024;23(1):416. 39716258 10.1186/s12944-024-02412-6 PMC11664894

[8] Xu R, Hu X, Wang T, Yang Y, Jiang N, Luo J, et al. Visceral Adiposity and Risk of Stroke: A Mendelian Randomization Study. Front Neurol. 2022;13: 804851. 35481268 10.3389/fneur.2022.804851 PMC9035635

[9] Lee V, Han Y, Toh DF, Bryant JA, Boubertakh R, Le TT, et al. Differential association of abdominal, liver, and epicardial adiposity with anthropometry, diabetes, and cardiac remodeling in Asians. Front Endocrinol (Lausanne). 2024;15:1439691. 39257902 10.3389/fendo.2024.1439691 PMC11385302

[10] Thomas DM, Bredlau C, Bosy-Westphal A, Mueller M, Shen W, Gallagher D, et al. Relationships between body roundness with body fat and visceral adipose tissue emerging from a new geometrical model. Obesity (Silver Spring). 2013;21(11):2264–71. 23519954 10.1002/oby.20408 PMC3692604

[11] Feng C, Lu C, Chen K, Song B, Shan Z, Teng W. Associations between various anthropometric indices and hypertension and hyperlipidaemia: a cross-sectional study in China. BMC Public Health. 2024;24(1):3045. 39497061 10.1186/s12889-024-20505-w PMC11536874

[12] Zhao Q, Zhang K, Li Y, Zhen Q, Shi J, Yu Y, et al. Capacity of a body shape index and body roundness index to identify diabetes mellitus in Han Chinese people in Northeast China: a cross-sectional study. Diabet Med. 2018;35(11):1580–7. 30059165 10.1111/dme.13787

[13] Li Y, He Y, Yang L, Liu Q, Li C, Wang Y, et al. Body Roundness Index and Waist-Hip Ratio Result in Better Cardiovascular Disease Risk Stratification: Results From a Large Chinese Cross-Sectional Study. Front Nutr. 2022;9: 801582. 35360688 10.3389/fnut.2022.801582 PMC8960742

[14] Zhang X, Ding L, Hu H, He H, Xiong Z, Zhu X. Associations of Body-Roundness Index and Sarcopenia with Cardiovascular Disease among Middle-Aged and Older Adults: Findings from CHARLS. J Nutr Health Aging. 2023;27(11):953–9.37997715 10.1007/s12603-023-2001-2

[15] Tong Y, Xu S, Huang L, Chen C. Obesity and insulin resistance: pathophysiology and treatment. Drug Discov Today. 2022;27(3):822–30. 34767960 10.1016/j.drudis.2021.11.001

[16] Cui C, Liu L, Qi Y, Han N, Xu H, Wang Z, et al. Joint association of TyG index and high sensitivity C-reactive protein with cardiovascular disease: a national cohort study. Cardiovasc Diabetol. 2024;23(1):156. 38715129 10.1186/s12933-024-02244-9 PMC11077847

[17] Zhang R, Hong J, Wu Y, Lin L, Chen S, Xiao Y. Joint association of triglyceride glucose index (TyG) and a body shape index (ABSI) with stroke incidence: a nationwide prospective cohort study. Cardiovasc Diabetol. 2025;24(1):7. 39762919 10.1186/s12933-024-02569-5 PMC11705842

[18] Cui C, Liu L, Zhang T, Fang L, Mo Z, Qi Y, et al. Triglyceride-glucose index, renal function and cardiovascular disease: a national cohort study. Cardiovasc Diabetol. 2023;22(1):325. 38017519 10.1186/s12933-023-02055-4 PMC10685637

[19] Zhao Y, Hu Y, Smith JP, Strauss J, Yang G. Cohort profile: the China Health and Retirement Longitudinal Study (CHARLS). Int J Epidemiol. 2014;43(1):61–8. 23243115 10.1093/ije/dys203 PMC3937970

[20] Yang M, Liu J, Shen Q, Chen H, Liu Y, Wang N, et al. Body Roundness Index Trajectories and the Incidence of Cardiovascular Disease: Evidence From the China Health and Retirement Longitudinal Study. J Am Heart Assoc. 2024;13(19): e034768. 39319466 10.1161/JAHA.124.034768 PMC11681446

[21] American Diabetes Association Professional Practice Committee. Diagnosis and Classification of Diabetes. Standards of Care in Diabetes-2024. Diabetes Care. 2024;47(Suppl 1):S20-s42. 38078589 10.2337/dc24-S002 PMC10725812

[22] Williams B, Mancia G, Spiering W, Agabiti Rosei E, Azizi M, Burnier M, et al. 2018 ESC/ESH Guidelines for the management of arterial hypertension. Eur Heart J. 2018;39(33):3021–104. 30165516 10.1093/eurheartj/ehy339

[23] Zengwu W, Jianjun L, Shuiping Z, Runlin G. Chinese guideline for lipid management (primary care version 2024). Zhonghua Xin Xue Guan Bing Za Zhi. 2024;52(4):330–7. 38548600 10.3760/cma.j.cn112148-20240102-00002

[24] de Jager DJ, de Mutsert R, Jager KJ, Zoccali C, Dekker FW. Reporting of Interaction. Nephron Clin Pract. 2011;119(2):c158–61. 21757954 10.1159/000327598

[25] Guerrero-Romero F, Simental-Mendía LE, González-Ortiz M, Martínez-Abundis E, Ramos-Zavala MG, Hernández-González SO, et al. The product of triglycerides and glucose, a simple measure of insulin sensitivity Comparison with the euglycemic-hyperinsulinemic clamp. J Clin Endocrinol Metab. 2010;95(7):3347–51. 20484475 10.1210/jc.2010-0288

[26] Yang Y, Huang X, Wang Y, Leng L, Xu J, Feng L, et al. The impact of triglyceride-glucose index on ischemic stroke: a systematic review and meta-analysis. Cardiovasc Diabetol. 2023;22(1):2. 36609319 10.1186/s12933-022-01732-0 PMC9825038

[27] Yan F, Yan S, Wang J, Cui Y, Chen F, Fang F, et al. Association between triglyceride glucose index and risk of cerebrovascular disease: systematic review and meta-analysis. Cardiovasc Diabetol. 2022;21(1):226. 36324146 10.1186/s12933-022-01664-9 PMC9632026

[28] Cai W, Xu J, Wu X, Chen Z, Zeng L, Song X, et al. Association between triglyceride-glucose index and all-cause mortality in critically ill patients with ischemic stroke: analysis of the MIMIC-IV database. Cardiovasc Diabetol. 2023;22(1):138. 37312120 10.1186/s12933-023-01864-x PMC10262584

[29] Miao M, Bi Y, Hao L, Bao A, Sun Y, Du H, et al. Triglyceride-glucose index and short-term functional outcome and in-hospital mortality in patients with ischemic stroke. Nutr Metab Cardiovasc Dis. 2023;33(2):399–407. 36586773 10.1016/j.numecd.2022.11.004

[30] Guo W, Liu Z, Liu P, Lu Q, Chang Q, Zhang M, et al. Association between triglyceride-glucose index and 1-year recurrent stroke after acute ischemic stroke: results from the Xi’an stroke registry study of China. Cerebrovasc Dis. 2024;53(4):391–402. 37757755 10.1159/000534240

[31] Chen Y, Yang Z, Liu Y, Li Y, Zhong Z, McDowell G, et al. Exploring the prognostic impact of triglyceride-glucose index in critically ill patients with first-ever stroke: insights from traditional methods and machine learning-based mortality prediction. Cardiovasc Diabetol. 2024;23(1):443. 39695656 10.1186/s12933-024-02538-y PMC11658255

[32] Grandl G, Wolfrum C. Hemostasis, endothelial stress, inflammation, and the metabolic syndrome. Semin Immunopathol. 2018;40(2):215–24. 29209827 10.1007/s00281-017-0666-5 PMC5809518

[33] Love KM, Barrett EJ, Malin SK, Reusch JEB, Regensteiner JG, Liu Z. Diabetes pathogenesis and management: the endothelium comes of age. J Mol Cell Biol. 2021;13(7):500–12. 33787922 10.1093/jmcb/mjab024 PMC8530521

[34] Hill MA, Yang Y, Zhang L, Sun Z, Jia G, Parrish AR, et al. Insulin resistance, cardiovascular stiffening and cardiovascular disease. Metabolism. 2021;119: 154766. 33766485 10.1016/j.metabol.2021.154766

[35] Li X, Weber NC, Cohn DM, Hollmann MW, DeVries JH, Hermanides J, et al. Effects of hyperglycemia and diabetes mellitus on coagulation and hemostasis. J Clin Med. 2021;10(11):2419. 34072487 10.3390/jcm10112419 PMC8199251

[36] Yoshida D, Ikeda S, Shinohara K, Kazurayama M, Tanaka S, Yamaizumi M, et al. Triglyceride-glucose index associated with future renal function decline in the general population. J Gen Intern Med. 2024;39(16):3225–33. 38782808 10.1007/s11606-024-08809-4 PMC11618565

[37] Shi S, Song Y, Liu Z, He J, Zheng Z, Song C, et al. The association of the triglyceride-glucose index with the risk of atrial fibrillation: Analysis of the UK Biobank. Nutr Metab Cardiovasc Dis. 2025;35(4): 103826. 39799098 10.1016/j.numecd.2024.103826

[38] Dang K, Wang X, Hu J, Zhang Y, Cheng L, Qi X, et al. The association between triglyceride-glucose index and its combination with obesity indicators and cardiovascular disease: NHANES 2003–2018. Cardiovasc Diabetol. 2024;23(1):8. 38184598 10.1186/s12933-023-02115-9 PMC10771672

[39] Guo D, Wu Z, Xue F, Chen S, Ran X, Zhang C, et al. Association between the triglyceride-glucose index and impaired cardiovascular fitness in non-diabetic young population. Cardiovasc Diabetol. 2024;23(1):39. 38245734 10.1186/s12933-023-02089-8 PMC10800072

[40] Gersh F, O’Keefe JH, Elagizi A, Lavie CJ, Laukkanen JA. Estrogen and cardiovascular disease. Prog Cardiovasc Dis. 2024;84:60–7. 38272338 10.1016/j.pcad.2024.01.015

[41] Sun Q, Yu D, Fan J, Yu C, Guo Y, Pei P, et al. Healthy lifestyle and life expectancy at age 30 years in the Chinese population: an observational study. Lancet Public Health. 2022;7(12):e994–1004. 35926549 10.1016/S2468-2667(22)00110-4 PMC7615002

[42] Shi Q, Nong K, Vandvik PO, Guyatt GH, Schnell O, Rydén L, et al. Benefits and harms of drug treatment for type 2 diabetes: systematic review and network meta-analysis of randomised controlled trials. BMJ. 2023;381: e074068. 37024129 10.1136/bmj-2022-074068 PMC10077111

[43] Rahimi K, Bidel Z, Nazarzadeh M, Copland E, Canoy D, Ramakrishnan R, Davis BR. Pharmacological blood pressure lowering for primary and secondary prevention of cardiovascular disease across different levels of blood pressure: an individual participant-level data meta-analysis. Lancet. 2021;397(10285):1625–36. 33933205 10.1016/S0140-6736(21)00590-0 PMC8102467

[44] Bibbins-Domingo K, Grossman DC, Curry SJ, Davidson KW, Epling JW Jr, García FAR, et al. Statin use for the primary prevention of cardiovascular disease in adults: US preventive services task force recommendation statement. JAMA. 2016;316(19):1997–2007. 27838723 10.1001/jama.2016.15450

[45] Wang P, Fan Y, Gao H, Wang B. Body roundness index as a predictor of all-cause and cardiovascular mortality in patients with diabetes and prediabetes. Diabetes Res Clin Pract. 2025;219: 111958. 39675484 10.1016/j.diabres.2024.111958

[46] Firouzi F, Ramezani Tehrani F, Kaveh A, Mousavi M, Azizi F, Behboudi-Gandevani S. Adiposity trajectories and cardiovascular disease risk in women: a population-based cohort study with a focus on menopausal status. Front Endocrinol (Lausanne). 2024;15:1389330. 38854691 10.3389/fendo.2024.1389330 PMC11157004

[47] Preda A, Carbone F, Tirandi A, Montecucco F, Liberale L. Obesity phenotypes and cardiovascular risk: from pathophysiology to clinical management. Rev Endocr Metab Disord. 2023;24(5):901–19. 37358728 10.1007/s11154-023-09813-5 PMC10492705

[48] Rana MN, Neeland IJ. Adipose tissue inflammation and cardiovascular disease: an update. Curr Diab Rep. 2022;22(1):27–37. 35179694 10.1007/s11892-021-01446-9

[49] Ren Y, Zhao H, Yin C, Lan X, Wu L, Du X, et al. Adipokines, hepatokines and myokines: focus on their role and molecular mechanisms in adipose tissue inflammation. Front Endocrinol (Lausanne). 2022;13: 873699. 35909571 10.3389/fendo.2022.873699 PMC9329830

[50] Yao F, Cui J, Shen Y, Jiang Y, Li Y, Liu X, et al. Evaluating a new obesity indicator for stroke risk prediction: comparative cohort analysis in rural settings of two nations. BMC Public Health. 2024;24(1):3301. 39605023 10.1186/s12889-024-20631-5 PMC11600789

[51] Shulman GI. Ectopic fat in insulin resistance, dyslipidemia, and cardiometabolic disease. N Engl J Med. 2014;371(23):2237–8. 25470706 10.1056/NEJMc1412427

[52] Fabbrini E, Magkos F, Mohammed BS, Pietka T, Abumrad NA, Patterson BW, et al. Intrahepatic fat, not visceral fat, is linked with metabolic complications of obesity. Proc Natl Acad Sci U S A. 2009;106(36):15430–5. 19706383 10.1073/pnas.0904944106 PMC2741268

[53] Wagner R, Eckstein SS, Yamazaki H, Gerst F, Machann J, Jaghutriz BA, et al. Metabolic implications of pancreatic fat accumulation. Nat Rev Endocrinol. 2022;18(1):43–54. 34671102 10.1038/s41574-021-00573-3

[54] Han Y, Sun Q, Chen W, Gao Y, Ye J, Chen Y, et al. New advances of adiponectin in regulating obesity and related metabolic syndromes. J Pharm Anal. 2024;14(5): 100913. 38799237 10.1016/j.jpha.2023.12.003 PMC11127227

[55] Saltiel AR, Olefsky JM. Inflammatory mechanisms linking obesity and metabolic disease. J Clin Invest. 2017;127(1):1–4. 28045402 10.1172/JCI92035 PMC5199709

[56] Szukiewicz D. Molecular mechanisms for the vicious cycle between insulin resistance and the inflammatory response in obesity. Int J Mol Sci. 2023;24(12):9818. 37372966 10.3390/ijms24129818 PMC10298329

[57] Huo RR, Liao Q, Zhai L, You XM, Zuo YL. Interacting and joint effects of triglyceride-glucose index (TyG) and body mass index on stroke risk and the mediating role of TyG in middle-aged and older Chinese adults: a nationwide prospective cohort study. Cardiovasc Diabetol. 2024;23(1):30. 38218819 10.1186/s12933-024-02122-4 PMC10790273

[58] Yang Y, Li S, Ren Q, Qiu Y, Pan M, Liu G, et al. The interaction between triglyceride-glucose index and visceral adiposity in cardiovascular disease risk: findings from a nationwide Chinese cohort. Cardiovasc Diabetol. 2024;23(1):427. 39604987 10.1186/s12933-024-02518-2 PMC11603997

[59] Kuk JL, Saunders TJ, Davidson LE, Ross R. Age-related changes in total and regional fat distribution. Ageing Res Rev. 2009;8(4):339–48. 19576300 10.1016/j.arr.2009.06.001

[60] Saisho Y, Butler AE, Meier JJ, Monchamp T, Allen-Auerbach M, Rizza RA, et al. Pancreas volumes in humans from birth to age one hundred taking into account sex, obesity, and presence of type-2 diabetes. Clin Anat. 2007;20(8):933–42. 17879305 10.1002/ca.20543 PMC2680737

[61] Shrestha S, Erikson G, Lyon J, Spigelman AF, Bautista A, Manning Fox JE, et al. Aging compromises human islet beta cell function and identity by decreasing transcription factor activity and inducing ER stress. Sci Adv. 2022;8(40):eabo3932. 36197983 10.1126/sciadv.abo3932 PMC9534504

[62] Tudurí E, Soriano S, Almagro L, Montanya E, Alonso-Magdalena P, Nadal Á, et al. The pancreatic β-cell in ageing: Implications in age-related diabetes. Ageing Res Rev. 2022;80: 101674. 35724861 10.1016/j.arr.2022.101674

