# Supplementary material for: Joint association of triglyceride glucose index (TyG) and body roundness index (BRI) with stroke incidence: a national cohort study
Source: Cardiovasc Diabetol. 2025 Apr 16;24:164. doi: 10.1186/s12933-025-02724-6 (PMC12004739; doi:10.1186/s12933-025-02724-6)
Supplement: Supplementary file 1 — Supplementary material 1 [file 12933_2025_2724_MOESM1_ESM.docx]

**Supplementary Materials**

**Table S1** The proportion of missing covariates and imputation methods

**Table S2** Baseline characteristics stratified by the median value of TyG index (median = 8.58)

**Table S3** Baseline characteristics stratified by the median value of BRI (median = 4.05)

**Table S4** Main effects of TyG and BRI considering the presence of interaction terms

**Table S5** Sensitivity analysis of the association between TyG and BRI in relation to stroke risk

**Fig. S1** Predictive capacity of the TyG index and BRI for stroke risk

**Fig. S2** Subgroup analysis for the effect of the TyG index on stroke risk

**Fig. S3** Subgroup analysis for the effect of the BRI on stroke risk

| **Table S1** The proportion of missing covariates and imputation methods | | | |
| --- | --- | --- | --- |
| Variable | Number of missing data | Percentage of missing data (%) | Imputation methods |
| Smoking | 10 | 0.15 | logreg |
| Drinking | 4 | 0.06 | logreg |
| BMI | 15 | 0.23 | pmm |
| SBP | 69 | 1.04 | pmm |
| DBP | 71 | 1.07 | pmm |
| Antihyperglycemic treatment | 61 | 0.92 | logreg |
| Antihypertensive treatment | 38 | 0.57 | logreg |
| Lipid-lowering treatment | 138 | 2.08 | logreg |
| Diabetes | 89 | 1.34 | logreg |
| Hypertension | 63 | 0.95 | pmm |
| Hyperlipemia | 78 | 1.18 | logreg |
| Kidney disease | 35 | 0.53 | logreg |
| Heart disease | 29 | 0.44 | logreg |
| LDL-C | 14 | 0.21 | pmm |
| Abbreviations: BMI: body mass index; DBP: diastolic blood pressure; LDL-C: low-density lipoprotein cholesterol; SBP: systolic blood pressure. | | | |

| **Table S2** Baseline characteristics stratified by the median value of TyG index (median = 8.58) | | | |
| --- | --- | --- | --- |
| Variables | TyG <median | TyG ≥median | P value |
| Number of participants | 3310 | 3311 |  |
| Age, years, mean (SD) | 58.0 (8.7) | 58.2 (8.4) | 0.423 |
| Male, n (%) | 1621 (49.0) | 1330 (40.2) | < 0.001 |
| Residence, n (%) |  |  | < 0.001 |
| Rural | 982 (29.7) | 1246 (37.6) |  |
| City | 2328 (70.3) | 2065 (62.4) |  |
| Marital status, n (%) |  |  | 0.551 |
| Married and living with spouse | 492 (14.9) | 474 (14.3) |  |
| Others | 2818 (85.1) | 2837 (85.7) |  |
| Education level, n (%) |  |  | 0.772 |
| Junior high school and below | 3001 (90.7) | 2994 (90.4) |  |
| Senior high school and above | 309 (9.3) | 317 (9.6) |  |
| Smoking, n (%) | 1330 (40.2) | 1120 (33.9) | < 0.001 |
| Drinking, n (%) | 1357 (41.0) | 1182 (35.7) | < 0.001 |
| BMI, kg/m^2^ | 22.3 (20.3, 24.4) | 24.37 (22.0, 27.0) | < 0.001 |
| SBP, mmHg | 123.3 (112.0, 137.3) | 129.3 (116.7, 144.0) | < 0.001 |
| DBP, mmHg | 73.0 (66.0, 81.3) | 76.3 (69.0, 84.7) | < 0.001 |
| Antihyperglycemic treatment, n (%) | 49 (1.5) | 176 (5.4) | <0.001 |
| Antihypertensive treatment, n (%) | 409 (12.4) | 803 (24.4) | < 0.001 |
| Lipid-lowering treatment, n (%) | 87 (2.7) | 236 (7.3) | < 0.001 |
| Diabetes, n (%) | 191 (5.9) | 801 (24.5) | < 0.001 |
| Hypertension, n (%) | 1050 (32.1) | 1570 (47.8) | < 0.001 |
| Hyperlipemia, n (%) | 750 (23.0) | 2392 (72.9) | < 0.001 |
| Kidney disease, n (%) | 188 (5.7) | 166 (5.0) | 0.256 |
| Heart disease, n (%) | 272 (8.2) | 429 (13.0) | < 0.001 |
| TC, mg/dL | 183.6 (163.2, 205.7) | 200.3 (176.7, 226.2) | < 0.001 |
| TG, mg/dL | 74.3 (60.2, 89.4) | 150.5 (123.9, 204.0) | <0.001 |
| HDL-C, mg/dL | 56.1 (47.9, 66.5) | 43.7 (36.3, 52.2) | < 0.001 |
| LDL-C, mg/dL | 112.5 (94.3, 133.0) | 119.9 (96.7, 143.8) | < 0.001 |
| UA, mg/dL | 4.1 (3.4, 4.9) | 4.4 (3.7, 5.3) | < 0.001 |
| CRP, mg/L | 0.8 (0.5, 1.7) | 1.2 (0.6, 2.3) | < 0.001 |
| BRI | 3.66 (2.97, 4.58) | 4.53 (3.61, 5.47) | < 0.001 |
| TyG | 8.22 (7.99, 8.41) | 9.01 (8.77, 9.38) | < 0.001 |
| Stroke, n (%) | 289 (8.7) | 454 (13.7) | < 0.001 |
| Values are mean (SD), median (IQR), or n (%). Abbreviations: BRI: body roundness index; BMI: body mass index; CRP: C-reactive protein; DBP: diastolic blood pressure; HDL-C: high-density lipoprotein cholesterol; IQR: Interquartile range; LDL-C: low-density lipoprotein cholesterol; SBP: systolic blood pressure; SD: standard deviation; TC: total cholesterol; TG: triglycerides; TyG: triglyceride glucose; UA: uric acid. | | | |

| **Table S3** Baseline characteristics stratified by the median value of BRI (median = 4.05) | | | |
| --- | --- | --- | --- |
| Variables | BRI <median | BRI ≥median | P value |
| Number of participants | 3310 | 3311 |  |
| Age, years, mean (SD) | 57.63 (8.45) | 58.50 (8.66) | < 0.001 |
| Male, n (%) | 1902 (57.5) | 1049 (31.7) | < 0.001 |
| Residence, n (%) |  |  | < 0.001 |
| Rural | 957 (28.9) | 1271 (38.4) |  |
| City | 2353 (71.1) | 2040 (61.6) |  |
| Marital status, n (%) |  |  | 0.706 |
| Married and living with spouse | 477 (14.4) | 489 (14.8) |  |
| Others | 2833 (85.6) | 2822 (85.2) |  |
| Education level, n (%) |  |  | 0.039 |
| Junior high school and below | 2972 (89.8) | 3023 (91.3) |  |
| Senior high school and above | 338 (10.2) | 288 ( 8.7) |  |
| Smoking, n (%) | 1567 (47.4) | 883 (26.7) | < 0.001 |
| Drinking, n (%) | 1497 (45.2) | 1042 (31.5) | < 0.001 |
| BMI, kg/m^2^ | 21.2 (19.7, 22.7) | 25.7 (23.8, 27.7) | < 0.001 |
| SBP,mmHg | 121.7 (111.0, 135.0) | 131.0 (118.0, 146.0) | < 0.001 |
| DBP, mmHg | 72.3 (65.3, 80.3) | 77.0 (69.7, 85.3) | < 0.001 |
| Antihyperglycemic treatment, n (%) | 61 (1.9) | 164 (5.0) | < 0.001 |
| Antihypertensive treatment, n (%) | 344 (10.5) | 868 (26.3) | < 0.001 |
| Lipid-lowering treatment, n (%) | 64 (2.0) | 259 (8.0) | < 0.001 |
| Diabetes, n (%) | 342 (10.5) | 650 (19.9) | < 0.001 |
| Hypertension, n (%) | 924 (28.2) | 1696 (51.6) | < 0.001 |
| Hyperlipemia, n (%) | 1187 (36.4) | 1955 (59.6) | <0.001 |
| Kidney disease, n (%) | 179 (5.4) | 175 (5.3) | 0.842 |
| Heart disease, n (%) | 264 (8.0) | 437 (13.2) | < 0.001 |
| TC, mg/dL | 187.3 (165.1, 210.7) | 196.0 (173.2, 222.1) | < 0.001 |
| TG, mg/dL | 88.5 (65.5, 129.2) | 120.4 (86.7, 177.0) | < 0.001 |
| HDL-C, mg/dL | 53.5 (44.1, 63.8) | 46.0 (38.3, 55.3) | < 0.001 |
| LDL-C, mg/dL | 112.1 (92.4, 132.6) | 120.2 (98.2, 143.4) | < 0.001 |
| UA, mg/dL | 4.2 (3.5, 5.0) | 4.3 (3.6, 5.1) | 0.025 |
| CRP, mg/L | 0.8 (0.5, 1.6) | 1.2 (0.7, 2.4) | < 0.001 |
| BRI | 3.24 (2.76, 3.64) | 5.08 (4.55, 5.84) | < 0.001 |
| TyG | 8.41 (8.09, 8.80) | 8.76 (8.40, 9.19) | < 0.001 |
| Stroke, n (%) | 270 (8.2) | 473 (14.3) | < 0.001 |
| Values are expressed as mean (SD), median (IQR), or n (%). Abbreviations: BRI: body roundness index; BMI: body mass index; CRP: C-reactive protein; DBP: diastolic blood pressure; HDL-C: high-density lipoprotein cholesterol; IQR: Interquartile range; LDL-C: low-density lipoprotein cholesterol; SBP: systolic blood pressure; SD: standard deviation; TC: total cholesterol; TG: triglycerides; TyG: triglyceride glucose; UA: uric acid. | | | |

| **Table S4** Main effects of TyG and BRI considering the presence of interaction terms | | | | | | |
| --- | --- | --- | --- | --- | --- | --- |
|  | Model 1 | | Model 2 | | Model 3 | |
|  | HR (95% CI) | P value | HR (95% CI) | P value | HR (95% CI) | P value |
| Low Tyg | Ref |  | Ref |  | Ref |  |
| High Tyg | 1.64 (1.29–2.09) | < 0.001 | 1.56 (1.22–1.98) | < 0.001 | 1.36 (1.05–1.75) | 0.018 |
| Low BRI | Ref |  | Ref |  | Ref |  |
| High BRI | 1.95 (1.54–2.47) | < 0.001 | 1.78 (1.40–2.25) | < 0.001 | 1.61 (1.27–2.05) | < 0.001 |

Model 1: adjusted for age and sex
Model 2: adjusted for variables in Model 1 plus smoking, drinking, marital status, education level, residence, and SBP
Model 3: adjusted for variables in Model 2 plus heart disease, diabetes, hypertension, hyperlipemia, kidney disease, C-reactive protein, and uric acid.

| **Table S5** Sensitivity analysis of the association between TyG and BRI in relation to stroke risk | | | | |
| --- | --- | --- | --- | --- |
|  | case | Incidence rate^a^  (95% CI) | Unadjusted models | Adjusted models |
|  |  |  | HR (95% CI), P value | HR (95% CI), P value |
| Excluding participants with missing covariate data | | | | |
| Low BRI | 261 | 9.4 (8.3–10.7) | Ref |  |
| High BRI | 450 | 16.5 (15.1–18.1) | 1.79 (1.53–2.08), <0.001 | 1.43 (1.21–1.70), <0.001 |
| Low TyG | 278 | 10.1 (8.9–11.3) | Ref |  |
| High TyG | 433 | 15.9 (14.5–17.5) | 1.60 (1.38–1.86), <0.001 | 1.23 (1.03–1.48), 0.023 |
| Low TyG and low BRI | 136 | 7.8 (6.6–9.3) | Ref |  |
| High TyG and low BRI | 125 | 12.1 (10.1–14.5) | 1.57 (1.23–2.00), <0.001 | 1.35 (1.04–1.75), 0.024 |
| Low TyG and high BRI | 142 | 13.8 (11.7–16.3) | 1.80 (1.42–2.27), <0.001 | 1.59 (1.24–2.03), <0.001 |
| High TyG and high BRI | 308 | 18.2 (16.2–20.3) | 2.39 (1.95–2.92), <0.001 | 1.74 (1.36–2.22), <0.001 |
| Excluding participants receiving antihyperglycemic treatment or antihypertensive treatment or lipid-lowering treatment | | | | |
| Low BRI | 176 | 7.7 (6.6–9.0) | Ref |  |
| High BRI | 273 | 12.1 (10.7–13.6) | 1.59 (1.31–1.92), <0.001 | 1.46 (1.19–1.80), <0.001 |
| Low TyG | 185 | 8.1 (7.0–9.4) | Ref |  |
| HighTyG | 264 | 11.7 (10.3–13.2) | 1.45 (1.21–1.75), <0.001 | 1.28 (1.03–1.59), 0.029 |
| Low TyG and low BRI | 86 | 6.1 (4.9–7.6) | Ref |  |
| High TyG and low BRI | 90 | 10.2 (8.3–12.6) | 1.69 (1.26–2.27), 0.001 | 1.57 (1.15–2.15), 0.005 |
| Low TyG and high BRI | 99 | 11.3 (9.2–13.8) | 1.86 (1.40–2.49), <0.001 | 1.81 (1.35–2.45), <0.001 |
| High TyG and high BRI | 174 | 12.6 (10.8–14.6) | 2.09 (1.62–2.71), <0.001 | 1.87 (1.39–2.53), <0.001 |
| Excluding participants with follow duration less than two years | | | | |
| Low BRI | 261 | 9.0 (7.9–10.1) | Ref |  |
| High BRI | 448 | 15.6 (14.2–17.1) | 1.77 (1.52–2.06), <0.001 | 1.43 (1.21–1.69), <0.001 |
| Low TyG | 272 | 9.3 (8.3–10.5) | Ref |  |
| HighTyG | 437 | 15.2 (13.9–16.7) | 1.66 (1.42–1.93), <0.001 | 1.31 (1.09–1.57), 0.003 |
| Low TyG and low BRI | 135 | 7.4 (6.2–8.7) | Ref |  |
| High TyG and low BRI | 126 | 11.7 (9.8–14.0) | 1.61 (1.26–2.05), <0.001 | 1.41 (1.09–1.83), 0.010 |
| Low TyG and high BRI | 137 | 12.7 (10.7–15.0) | 1.75 (1.38–2.22), <0.001 | 1.55 (1.21–1.98), <0.001 |
| High TyG and high BRI | 311 | 17.3 (15.5–19.4) | 2.42 (1.98–2.96), <0.001 | 1.82 (1.43–2.32), <0.001 |
| Including participants with non-fasting blood samples at baseline | | | | |
| Low BRI | 296 | 9.2 (8.2–10.3) | Ref |  |
| High BRI | 518 | 16.3 (15.0–17.8) | 1.81 (1.57–2.09), <0.001 | 1.45 (1.24–1.70), <0.001 |
| Low TyG | 318 | 9.9 (8.8–11.0) | Ref |  |
| HighTyG | 496 | 15.6 (14.3–17.0) | 1.60 (1.39–1.85), <0.001 | 1.25 (1.06–1.48), 0.009 |
| Low TyG and low BRI | 153 | 7.6 (6.5–8.9) | Ref |  |
| High TyG and low BRI | 143 | 11.8 (10.0–14.0) | 1.57 (1.25–1.98), <0.001 | 1.36 (1.07–1.74), 0.013 |
| Low TyG and high BRI | 165 | 13.7 (11.7–16.0) | 1.83 (1.47–2.28), <0.001 | 1.60 (1.27–2.01), <0.001 |
| High TyG and high BRI | 353 | 17.9 (16.2–20.0) | 2.43 (2.01–2.93), <0.001 | 1.78 (1.42–2.24), <0.001 |
| ^a^ Per 1000 person-years.  Adjusted models: adjusted for age, sex, smoking, drinking, marital status, education level, residence, SBP, heart disease, diabetes, hypertension, hyperlipemia, kidney disease, C-reactive protein, and uric acid.  Abbreviations: BRI: body roundness index; TyG: triglyceride glucose. | | | | |


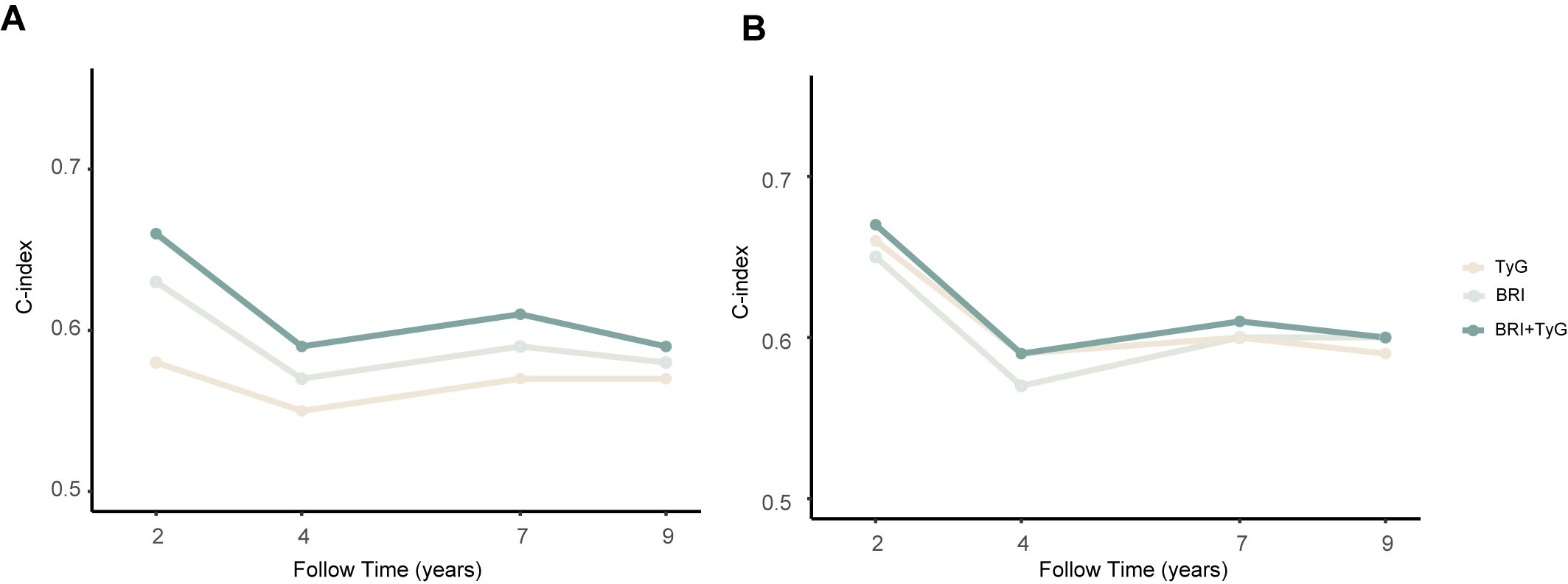


**Fig. S1** Predictive capacity of the TyG index and BRI on the stroke risk. (A) TyG and BRI as categorical variables; (B) TyG and BRI as continuous variables. Abbreviations: BRI: body roundness index; TyG: triglyceride glucose.


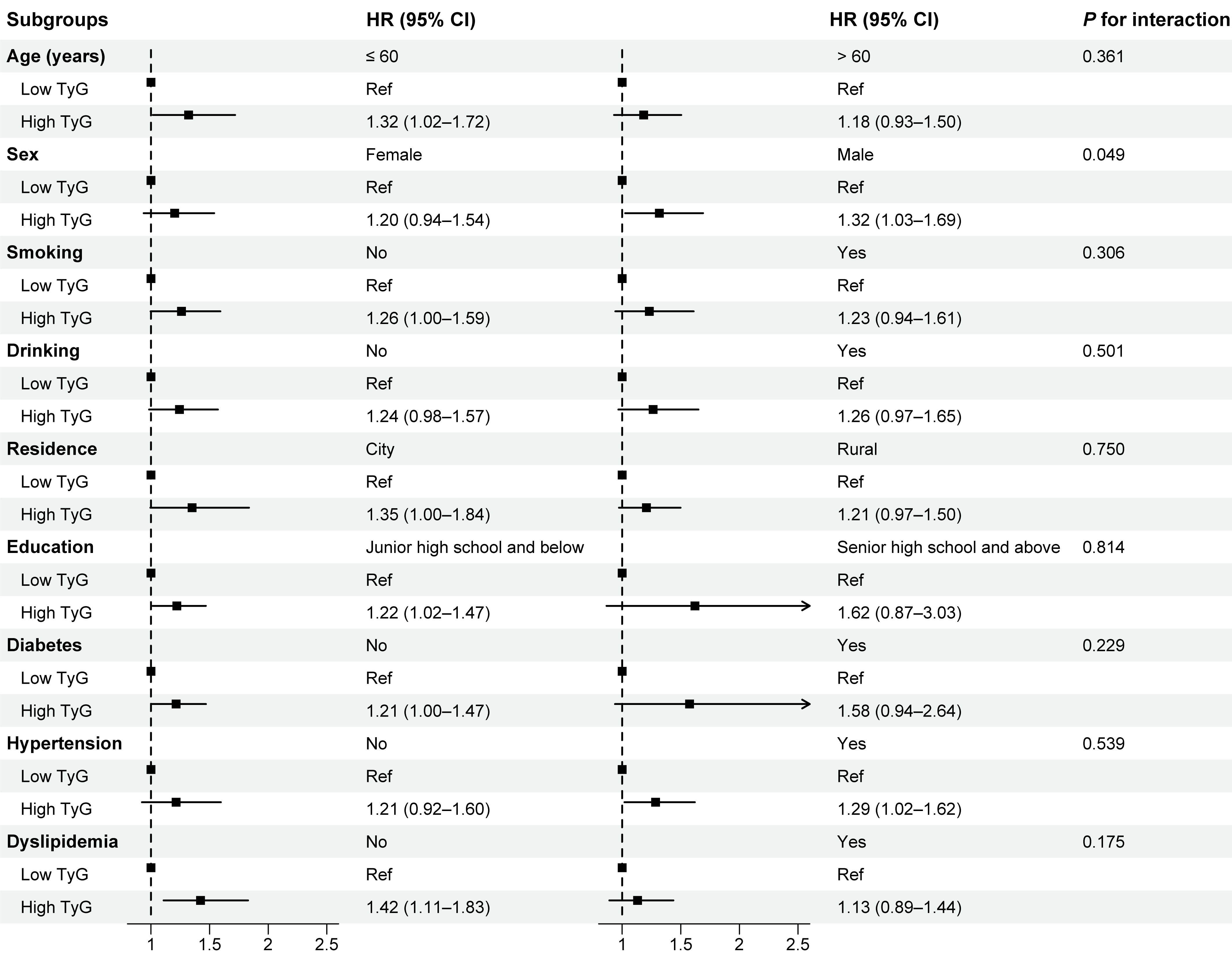


**Fig. S2** Subgroup analysis for the effect of the TyG index on stroke risk. Abbreviations: TyG: triglyceride glucose.


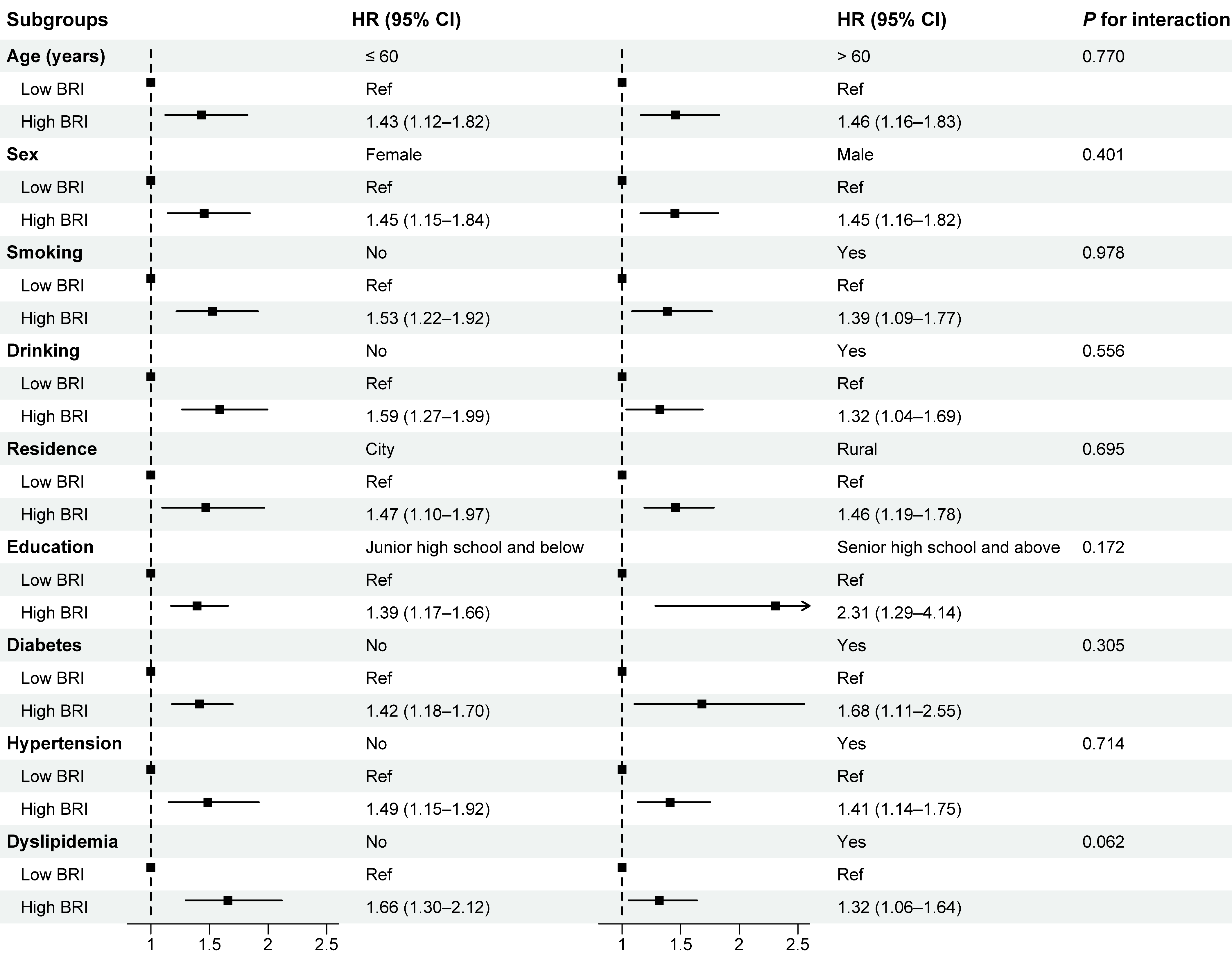


**Fig. S3** Subgroup analysis for the effect of the BRI on stroke risk. Abbreviations: BRI: body roundness index.
